# Medication-related suicide plans in children and adolescents: findings from crisis conversations

**DOI:** 10.1186/s40621-026-00691-4

**Published:** 2026-05-27

**Authors:** Ignacio J. Tripodi, Greg Buda, Margaret Meagher, Elizabeth A. Olson

**Affiliations:** Research & Impact, Crisis Text Line, 225 West 34th St, Floor 9-PMB # 9135, 10122-0901 New York, USA

**Keywords:** Suicide, Overdose, Adolescents

## Abstract

**Objectives:**

To identify medications frequently mentioned in suicide plans by texters at a crisis intervention service, and to evaluate age differences.

**Methods:**

We developed a natural language processing pipeline to extract medications mentioned in suicide plans. In conversations from 5,691 texters, we examined the frequency with which each medication was mentioned in texters under age 18 (*N* = 2,332) versus age 18 and older (*N* = 3,359).

**Results:**

The ten most frequently mentioned medications during discussions of planned suicide by overdose were: ibuprofen (16.5% of conversations), acetaminophen (16.1%), alprazolam (9.2%), diphenhydramine (7.3%), insulin (6.0%), melatonin (5.4%), sertraline (3.4%), aspirin (3.1%), fentanyl (3.0%), and trazodone (3.0%). Ibuprofen, acetaminophen, melatonin, and amphetamine were mentioned significantly more often by texters under age 18 than those 18 or older.

**Conclusions:**

Individuals under age 18 are particularly likely to plan suicide attempts using medications that are available over the counter, including ibuprofen and acetaminophen. In contrast with some prior reports, insulin and melatonin were frequently mentioned. The importance of public health measures including access restriction is emphasized.

**Supplementary Information:**

The online version contains supplementary material available at 10.1186/s40621-026-00691-4.

## Introduction

Self-poisoning by medication overdose is the most common suicide attempt method, resulting in significant morbidity and mortality in the United States annually [1]. Identifying which medications are used in suicide plans is critical for primary prevention efforts, including access restrictions. An analysis of National Poison Data System (NPDS) data found that, in adolescents ages 10–19, analgesics were the most common medication in suicide-related self-poisoning, followed by antidepressants, sedatives/ hyponotics/ antipsychotics, antihistamines, cough and cold preparations, anticonvulsants, stimulants and ‘street drugs’, cardiovascular drugs, antimicrobials, and alcohols [2]. Within the analgesic category, over the counter (OTC) medications accounted for 81.7% of cases, with opioids next, accounting for only 13.7% of cases [2]. Within this age group, suicide-related intentional overdose on OTC analgesics including ibuprofen and acetaminophen increased during the pandemic (2020–2022), along with attempts involving selective serotonin reuptake inhibitors (SSRIs: sertraline, fluoxetine), and diphenhydramine [3]. In a recent analysis of NPDS cases involving 10–25 year-olds from 2000 to 2018, 24.5% of incidents had a serious medical outcome [4], highlighting the impact of medication overdoses in this age group. Four times as many NPDS calls about adolescent suicide-related medication overdoses come from healthcare facilities versus the general public [3], meaning that the NPDS dataset predominantly reflects cases that became the target of medical attention. Less is known about medication-related suicide *plans* in adolescents.

The dataset used in this study is extracted from de-identified conversations at Crisis Text Line, a nonprofit organization that provides 24/7, free, confidential text-based crisis support to texters of all ages. The Crisis Text Line dataset provides an important source of public health surveillance data that differs from existing databases: this dataset captures information related to suicide plans or attempts that may or may not result in medical attention. For example, calls to poison control centers typically are placed only when a suicide attempt comes to the attention of family, friends, or medical personnel, or when the person attempting changes their mind. In combination with other sources, this crisis conversation dataset can therefore provide the field with a more comprehensive view of medication-related suicide plans, including plans in which the person does not seek or receive medical attention. We previously validated a natural language processing (NLP)/ machine learning (ML) pipeline to extract and classify mentions of planned suicide methods in crisis conversations, finding that overdose was the most common planned method [5]. In the present analysis, we extended this work to extract specific drug mentions in order to identify actionable targets for prevention. Given the relative scarcity of data related to suicide plans in children and adolescents, the possibility that age might affect method selection due to availability or familiarity, and the potential opportunities for age-specific interventions such as means restriction strategies, the principal aim of this study was to identify whether there were differences between texters over versus under age 18 in mentions of specific medications in suicide plans.

## Methods

We evaluated Crisis Text Line conversations that occurred between September 2017 and September 2024. Conversations were considered for inclusion if the conversation was in English and the volunteer crisis responder (VCR) indicated that the texter had mentioned a planned suicide method. As previously described [5], we built a classifier to detect the VCR asking about planned suicide methods (precision = 0.92, recall = 0.90, F1 = 0.91). We next compiled a custom medication lexicon (Supplement: medication lexicon), using a combination of regular expression matching and fuzzy matching to drug terms in FDA’s National Drug Code Directory [6], to detect medication mentions in the texter’s response. In cases where we did not detect the VCR asking about planned methods, we searched for overdose-related keywords (Supplement: overdose keyword list) in all of the texter’s messages, and then applied the custom medication lexicon to the messages where those keywords were detected. Because individual texters may reach out repeatedly with the same plan, we retained only the first conversation from each texter during this time period. Sterling IRB evaluated this research, for which it issued an exempt determination. Demographic data for texters who mentioned drug names and completed the optional post-conversation survey are available in the Supplement (Supplement Table 1).

### Demographic data

Demographic data are collected through an optional post-conversation survey, completed by approximately 20% of texters. For analyses involving texter age in the analytic sample, we used three sources: [1] this survey self-report, whenever available; or [2] extraction from conversation transcripts where age was mentioned; or if neither of those are available then [3] age assignment using a trained LASSO logistic regression model (see Supplement). For texters under age 18 who mentioned drug names, age was self-reported in 18.6% of texters (*N* = 433) and was imputed (via extraction or the LASSO model) for 81.4% of texters (*N* = 1,899). For texters 18+, age was self-reported in 13.0% of texters (*N* = 436) and was imputed in 87.0% of texters (*N* = 2,923). The distribution of self-reported vs. imputed age differed across age groups, Χ^2^(1) = 32.79, *p* < 0.001.

### Statistics

Statistical analyses were conducted in R version 4.4.2. We used Chi-square tests to assess age differences for each of the twenty most mentioned medications; tests were Bonferroni corrected for 20 comparisons (considered significant at *p* < 0.0025).

## Results

We detected 35,971 conversations about intentional overdose, 5,691 (15.8%) of which mentioned a specific drug name. The rest referred generically to overdose or to types of medication (e.g. ‘pain pills’, ‘some pills’, ‘my meds’) without naming specific drugs. 14.0% of texters under 18 mentioned specific drugs, while 17.4% of texters 18 or older mentioned specific drugs; this difference was statistically significant, Χ^2^(1) = 73.1, *p* < 0.0001. This dataset included conversations with *N* = 2,332 texters under 18 and *N* = 3,359 conversations with texters age 18 or older. Most texters (86.6%) mentioned just one medication, but some mentioned two (10.8%) or three or more (2.6%). Patterns of mentioning multiple drugs were comparable for texters under 18 (one: 88.3%; two: 10.3%; three or more: 1.4%) and texters 18 or older (one: 85.4%; two: 11.1%; three or more: 3.4%).

The twenty most frequently mentioned medications during discussions of planned suicide by overdose, in order of overall frequency, were: ibuprofen (16.5% of conversations), acetaminophen (16.1%), alprazolam (9.2%), diphenhydramine (7.3%), insulin (6.0%), melatonin (5.4%), sertraline (3.4%), aspirin (3.1%), fentanyl (3.0%), trazodone (3.0%), fluoxetine (2.6%), clonazepam (2.1%), lorazepam (1.9%), acetaminophen/ dextromethorphan (1.9%), amphetamine/ dextroamphetamine (1.5%), escitalopram (1.4%), acetaminophen/ oxycodone (1.4%), quetiapine (1.3%), lithium (1.2%), and zolpidem (1.2%).

### Age group differences

Ibuprofen, acetaminophen, melatonin, and amphetamine were mentioned significantly more often by texters under age 18 than those 18 or older (Table 1). In contrast, older texters mentioned alprazolam, insulin, fentanyl, trazodone, clonazepam, lorazepam, quetiapine, lithium, and zolpidem significantly more often than those under age 18.

**Table 1:** Top-20 most commonly mentioned medications in text-based conversations involving suicide plans by overdose

Drug name	Mentions under 18 (*N* = 2,332)	Mentions 18 or older (*N* = 3,359)	Overall mentions (*N* = 5691)	X^2^	*p*-value	Cramer’s V
1. Ibuprofen	**620 (26.6%)**	319 (9.5%)	939 (16.5%)	290.54	< 0.0001 *	0.226
2. Acetaminophen	**464 (19.9%)**	452 (13.5%)	916 (16.1%)	41.80	< 0.0001 *	0.086
3. Alprazolam	160 (6.9%)	**363 (10.8%)**	523 (9.2%)	25.21	< 0.0001 *	0.067
4. Diphenhydramine	175 (7.5%)	241 (7.2%)	416 (7.3%)	0.17	0.676	0.006
5. Insulin	72 (3.1%)	**269 (8.0%)**	341 (6.0%)	58.30	< 0.0001 *	0.101
6. Melatonin	**226 (9.7%)**	83 (2.5%)	309 (5.4%)	138.34	< 0.0001 *	0.156
7. Sertraline	95 (4.1%)	97 (2.9%)	192 (3.4%)	5.58	0.018	0.031
8. Aspirin	74 (3.2%)	102 (3.0%)	176 (3.1%)	0.05	0.83	0.003
9. Fentanyl	27 (1.2%)	**146 (4.4%)**	173 (3.0%)	46.41	< 0.0001 *	0.09
10. Trazodone	25 (1.1%)	**148 (4.4%)**	173 (3.0%)	50.78	< 0.0001 *	0.094
11. Fluoxetine	78 (3.3%)	70 (2.1%)	148 (2.6%)	8.15	0.004	0.038
12. Clonazepam	8 (0.3%)	**112 (3.3%)**	120 (2.1%)	58.23	< 0.0001 *	0.101
13. Lorazepam	12 (0.5%)	**97 (2.9%)**	109 (1.9%)	40.01	< 0.0001 *	0.084
14. Acetaminophen; dextromethorphan; phenylephrine (e.g., cold and flu medicine)	38 (1.6%)	70 (2.1%)	108 (1.9%)	1.29	0.256	0.015
15. Amphetamine; dextroamphetamine	**50 (2.1%)**	35 (1.0%)	85 (1.5%)	10.63	0.001*	0.043
16. Escitalopram	36 (1.5%)	46 (1.4%)	82 (1.4%)	0.18	0.668	0.006
17. Acetaminophen; oxycodone	26 (1.1%)	52 (1.6%)	78 (1.4%)	1.60	0.205	0.017
18. Quetiapine	7 (0.3%)	**66 (2.0%)**	73 (1.3%)	28.82	< 0.0001 *	0.071
19. Lithium	9 (0.4%)	**60 (1.8%)**	69 (1.2%)	21.38	< 0.0001 *	0.061
20. Zolpidem	3 (0.1%)	**66 (2.0%)**	69 (1.2%)	37.23	< 0.0001 *	0.081

* Significant age difference at Bonferroni-corrected *p* < 0.0025

Data are sorted by overall frequency of mentions by texters under 18 (*N* = 2,332) and 18 or older (*N* = 3,359) who mentioned overdose plans involving specific medications; age group differences between texters under age 18 versus 18 and up are noted. We reported on specific drug mentions, so if a texter mentioned three drugs, they are each included separately in the results table

## Discussion

The most commonly mentioned medications were acetaminophen and ibuprofen, consistent with existing literature [7–9]. To our knowledge, this is the first study to directly evaluate age differences in medication overdose suicide plans. Ibuprofen, acetaminophen, melatonin, and amphetamine were mentioned significantly more often by texters under age 18 than those 18 or older. Texters under age 18 were nearly 2 times as likely as texters 18 and older to plan to use ibuprofen, and they were also more likely to plan to use acetaminophen.

The remainder of the top 10 most-mentioned medications included alprazolam, diphenhydramine, insulin, melatonin, sertraline, aspirin, fentanyl, and trazodone. Unlike the remaining medications, there is less systematic literature regarding melatonin in suicide attempts [10]. Melatonin was a very common planned method in this dataset and was the *third* most commonly proposed medication in texters under age 18. Texters under age 18 were more than 2.5 times as likely to plan suicide via melatonin overdose as texters age 18 or older. Although melatonin has a low toxicity profile, the prevalence of this method in young texters is still concerning from a behavioral standpoint: prior suicide attempts predict subsequent attempts and may lower the threshold for attempting. Attempts via melatonin may go undetected by family and healthcare providers but may elevate the likelihood of subsequent attempts.

Our sample includes over 2,300 children and teenagers under age 18, an understudied population that shows unique patterns. About one third of these children were likely age 13 and under (Supplement). Study limitations include limited data regarding outcomes, because data are restricted to the time period during which the individual considers the attempt. Individuals who text crisis lines may differ from the overall population in important ways, including that they are generally help-seeking. Our analysis was restricted to medication overdoses: overdose on household products was a separate category in our original validation (‘Poisoning’), so the present study does not address non-medication overdose. It also is the case that out of 35,971 conversations, only 5,691 (15.8%) out of 35,971 conversations mentioned a specific medication name. It is possible that the analysis is biased toward a sample with deeper specific knowledge about medications, or possibly with more advanced/specific suicide plans. Further work to understand overdose plans in those who do not mention specific drugs could help reveal which medications are actually under consideration in that sample. Additionally, demographic data are available only for a subsample; we restricted our demographic analysis to age, for which we have validated imputation methods. Future work could help to identify drivers of selection of different drugs for planned overdose across other aspects of demographics such as gender and race. Our age model itself has been evaluated for bias, but because there was more self-reported age data in the younger group, a final limitation is that there is somewhat more uncertainty for the age assignments in the older sample than in the younger sample. Future work might examine changes in these patterns over time, particularly in response to social/ historical/ policy changes.

To summarize, in a large sample of text-based crisis conversations, we used an NLP/ML based extraction and classification strategy to evaluate medication mentions in suicide plans, finding significant age differences, including more frequent mentions of plans involving ibuprofen, acetaminophen, melatonin, and amphetamine by texters under age 18 than in those 18 or older. This study lays the groundwork for future NLP/ML-based research on unstructured suicide-related conversational data related to drug overdose, with critical implications for suicide prevention and public health.

### Public health implications

Individuals under age 18 are particularly likely to plan suicide attempts using medications that are available over the counter, including ibuprofen and acetaminophen. Age restrictions of OTC sales and pack size restrictions have been implemented in countries including the United Kingdom, Australia, and Denmark, and these have been associated with declines in poisonings [11].

Additionally, this study found that insulin and melatonin are frequently nominated as medications for intentional overdose. Relatives and healthcare providers should be aware that these may be target medications in suicide plans.

While public awareness of the need for safe firearm storage has grown, awareness of the need for safe medication storage has lagged [12]. Healthcare practitioners who treat individuals at risk for suicide may have particular roles to play in emphasizing safe storage of medications that may be involved in suicide attempts. Although further research is needed regarding factors that may affect the long-term effectiveness of these strategies, public health measures such as bottle labeling for these risks and widespread distribution of at-home medication lockboxes could also prevent harm and save lives.

## Electronic Supplementary Material

Supplementary Material 1

## Data Availability

The data will not be shared; because of the sensitive nature of the conversational dataset, we are not able to make these data publicly available even though they are de-identified. Crisis Text Line does invite selected academic research collaborations, and data may become available to selected collaborators through that program.
