# Supplementary material for: Medication-related suicide plans in children and adolescents: findings from crisis conversations
Source: Inj Epidemiol. 2026 May 27;13:53. doi: 10.1186/s40621-026-00691-4 (PMC13393860; doi:10.1186/s40621-026-00691-4)
Supplement: Supplementary file 1 — Supplementary Material 1 [file 40621_2026_691_MOESM1_ESM.docx]

**Supplemental** **Methods**

*Natural Language Processing (NLP): overdose keyword list*

We curated a list of keywords and linguistic patterns to detect mentions of planned suicide by medication overdose via regular expression matching, to capture self-reported overdose plans. This is needed because texters occasionally disclose plans prior to the VCC’s question about suicide methods (e.g., in the opening lines of the conversation).

These keywords included:

*od*

*overdose*

*overdosed*

*overdosing*

*overdosin*

*over dose*

*over dosed*

*over dosing*

*over dosin*

*overdoes*

*overdoas*

*over doas*

*overdoasing*

*over doasing*

*overdows*

*over dows*

*overdowsing*

*over dowsing*

*passing out forever*

*pass out forever*

*NLP: medication lexicon*

We compiled a custom lexicon of keywords for medication matching, starting with FDA’s National Drug Code Directory[^4^](https://www.zotero.org/google-docs/?IWsouz). We focused on substance names, as well as proprietary and non-proprietary names from this database. We built a library of common misspellings using texter messages where linguistic patterns of medication overdose were detected. We filtered words in these messages by part-of-speech (POS) tagging, to focus on nouns and adjectives and perform Levenshtein distance-based fuzzy matching between every word and every medication in our lexicon. Nearly 2,700 candidate terms were manually screened to map them to the correct medication name. Between generic drug mentions, misspellings, commercial and chemical drug names, we curated a total of 5,040 strings in our lexicon to identify medication mentions. For mentions of specific medications (rather than general categories e.g., “painkillers”), all results were harmonized to the specific chemical or mixture listed in the FDA’s National Drug Code (NDC) database. For example, all mentions of “tylenol”, “acetaminophen”, “paracetamol”, or “children’s acetaminophen”, were mapped to “acetaminophen”. These remapped NDC labels were used as the medication labels in the final analysis.

*Machine learning classifier for age detection*

To detect texter age when not provided in the post-conversation survey or the conversation text, we built a LASSO logistic regression model trained on over 390,000 conversations where the texters shared their age in the survey. The logistic regression model’s input features include a binary Bag of Words representation of the unigrams, bigrams, and trigrams in the texter’s messages, issue tags and ladder-up suicide assessment checkboxes annotated by the crisis counselor, discretized time of day, modality (e.g. SMS or webchat), binary indicators for mentions of words related to 12 coping mechanism groups, as well as standardized features such as the number of preceding conversations by the same texter, POS tag counts, messaging latency, conversation duration, word count, vocabulary size, count of uppercase words, count of common internet acronyms, count of emojis and emoticons, and letter lengthening. We performed parameter tuning of the LASSO penalty term using Bayesian search. For the binary task of predicting whether a texter is under 18 or 18+ years old, this method achieves 88.6% accuracy with an F1-score of 87.4%.

**Supplemental Results**

**Table 1**: Demographics for texters who reported complete demographic information on the post-conversation survey (N = 869)

| **Demographic Category** | **Specific Category** | **N** | **Percentage of applicable conversations** |
| --- | --- | --- | --- |
| Age (years) | 13 or younger | 141 | 16.2% |
|  | 14-17 | 292 | 33.6% |
|  | 18-24 | 199 | 22.9% |
|  | 25-34 | 121 | 13.9% |
|  | 35-44 | 49 | 5.6% |
|  | 45-54 | 42 | 4.8% |
|  | 55-64 | 22 | 2.5% |
|  | 65+ | 3 | 0.4% |
| Gender | Boy/Man only | 114 | 13.1% |
|  | Girl/Woman only | 558 | 64.2% |
|  | TGD | 172 | 19.8% |
| Race and ethnicity | Asian | 57 | 6.6% |
|  | Black / African American | 118 | 13.6% |
|  | Hispanic / Latinx | 135 | 15.5% |
|  | Middle Eastern / North African / Arab | 12 | 1.4% |
|  | Native American / Native Alaskan / Indigenous | 39 | 4.5% |
|  | Native Hawaiian / Pacific Islander | 11 | 1.3% |
|  | Other | 41 | 4.7% |
|  | White | 538 | 61.9% |

Demographics information for the subset of texters who mentioned a specific drug and self-reported age, gender and ethnicity (N=869). Race and ethnicity were assigned via a multiple selection item; percentages do not sum to 100 because texters could select multiple responses. TGD: transgender and gender diverse.
